# OA29 When IL-1 Inhibition is Just the Beginning: The Escalating Ladder of Refractory MAS.

**DOI:** 10.1093/rap/rkag113.029

**Published:** 2026-11-03

**Authors:** Sandhya Govindarajan, Ethan Sen, Sharmila Jandial, Flora McErlane, Sunil Sampath

**Affiliations:** Great North Children’s Hopsital, Newcastle, United Kingdom; Great North Children’s Hopsital, Newcastle, United Kingdom; Great North Children’s Hopsital, Newcastle, United Kingdom; Great North Children’s Hopsital, Newcastle, United Kingdom; Great North Children’s Hopsital, Newcastle, United Kingdom

## Abstract

**Introduction:**

Systemic Juvenile Idiopathic Arthritis (sJIA), a severe pediatric autoinflammatory disorder is often complicated by a life-threatening cytokine storm - overt Macrophage Activation Syndrome (MAS) in approximately 10% of patients and sub-clinical MAS in 30-40% patients [1]. While prompt initiation of high-dose intravenous corticosteroids serves as standard first-line management,emerging evidence shows only 14% of MAS responded to corticosteroids as monotherapy [2]. Despite modern advancements, MAS management remains a major challenge, especially when standard treatments fail.Therapeutic approaches and escalation hierarchy of treatment in MAS are still extremely variable, with potential significant discrepancies across different centers and countries.

**Case description:**

We report a paediatric patient presenting with a three-week history of quotidian fevers, arthralgia, evanescent urticaria-like rash, cervical lymphadenitis, and serositis, fulfilling PRINTO criteria for sJIA. Initial response to intravenous methylprednisolone was transient. The patient rapidly developed severe, refractory MAS characterised by haemodynamic collapse necessitating two Paediatric Intensive Care Unit (PICU) admissions with inotropic support, peak ferritin of 149,364 µg/L, acute liver injury, haemophagocytosis, coagulopathy, electrolyte derangement, prolonged QTc, elevated pro-BNP and troponin, and lower respiratory tract infection precipitating the second PICU admission. Whole-body MRI revealed widespread myositis extending from jaw to lower limb musculature, with normal CK and negative myositis-specific antibodies. Lung function testing demonstrated reduced TLCO (66% predicted after haemoglobin correction), raising concern for sJIA-associated interstitial lung disease. Elevated anti-GAD and ZnT8 antibodies prompted endocrine monitoring. Cytokine profiling confirmed markedly elevated IL-18, sCD25 and CXCL9, with normal cytotoxic granule exocytosis pathway studies, supporting secondary rather than primary HLH. Genetic panel (R15) identified no pathogenic variants.

**Management and Outcome:**

Two PICU admissions required invasive haemodynamic support with central access and dual inotropic support. Profound immunosuppression necessitated comprehensive prophylaxis: atovaquone (Pneumocystis jirovecii pneumonia), caspofungin transitioning to posaconazole (antifungal), and valaciclovir (antiviral). Recurrent electrolyte derangement required serial supplementation of phosphate (intravenous and oral), magnesium, potassium, calcium and sodium. Sequential immunosuppressive escalation encompassed high-dose corticosteroids, anakinra (up to 12 mg/kg/day intravenously), ciclosporin, IVIG, emapalumab, etoposide, and baricitinib, guided by regional and national MDT. Haematopoietic stem cell transplantation (HSCT) was held in reserve pending genetic results; tissue typing was performed and discussed with immunology, but was not required following sustained clinical and biochemical response to medical therapy and subsequent confirmation of negative genetics on R15 panel. Etoposide produced progressive biochemical remission with ferritin normalising to 475 µg/L at discharge. Secondary bone marrow failure (nadir neutrophils 0.02) resolved spontaneously.

**Discussion:**

Managing severe sJIA-associated MAS requires navigating deep therapeutic uncertainty. Frontline consensus supports empirical immunomodulation with corticosteroids, IVIG, and anakinra (∼10 mg/kg)[3], but escalation hierarchies vary widely globally. In the METAPHOR systematic review, which specifically identified anakinra use for sJIA across 5 of 17 analyzed cohort studies, severe cases required scaling anakinra to 11.9–15.4 mg/kg/day alongside corticosteroids, cyclosporine, and IVIG[2]. However, our patient failed these maximum medication combinations, necessitating an aggressive multi-tiered salvage protocol, forcing a rigid treatment escalation hierarchy:

1. Frontline: Three corticosteroid pulses and subcutaneous anakinra.

2. Intensification: High-dose intravenous anakinra, dual IVIG infusions, and cyclosporine.

3. Targeted Salvage: Emapalumab (anti-IFN-γ) and baricitinib (JAK1/2 inhibitor).

4. Cytotoxic Rescue: Etoposide induction (150 mg/m², twice weekly, 4 doses).

The novel bispecific antibody MAS825 was explicitly considered but ultimately deemed clinically redundant within the context ongoing, treatment-resistant signaling cascade. Diagnostic complexity peaked when a whole-body MRI revealed extensive pan-myositis, raising temporary concerns for an underlying primary muscle disease. However, comprehensive myositis antibodies processed from the pre-treatment sample were entirely negative, indicating pan-myositis as a rare manifestation of the hyperinflammatory cytokine storm itself. Invasive procedures (bone marrow, lumbar puncture, PET-CT) were considered but safely averted due to rapid clinical pivots.

A critical management dilemma emerged regarding emapalumab titration during an intercurrent chest infection-toggling between 3 mg/kg and 6 mg/kg (12 doses total) - highlighting the lack of international consensus on managing IFN-γ suppression during active viral signals, such as transiently positive HHV-6 PCRs. Furthermore, a clear lack of expert consensus exists regarding post-stabilisation weaning hierarchies, with highly differential opinions on whether to taper steroids or cyclosporine first. Ultimately, this comprehensive multidisciplinary approach allowed successful cyclosporine cessation, leaving the patient completely stable on maintenance baricitinib, anakinra, and cautious prednisolone wean with antimicrobial prophylaxis with ongoing rehabilitation.

**Key learning points:**

• **Pivoting the Molecular Target**: When severe MAS breaks through maximum extracellular IL-1 and IL-6 blockades, rapid escalation to combined intracellular JAK1/2 inhibition (**baricitinib**), targeted IFN-γ blockade (**emapalumab**), and cytotoxic destruction (**etoposide**) can resolve a multi-drug resistant storm.

• **Decoding Severe Phenotypes**: Explosive sJIA-MAS can present with rare clinical mimics like **extensive pan-myositis on whole-body MRI**. Securing **pre-treatment serum samples** is critical to rule out primary muscle overlap syndromes and avoid diagnostic delays.

• **Bridging the Consensus Gap**: Standardised international protocols are needed to guide biologic titration during secondary infections (under IFN-γ suppression) and to define optimal weaning sequences between medications such as steroids and cyclosporine.

**References**:

1. Minoia F, Davì S, Horne A, et al. Clinical features, treatment, and outcome of macrophage activation syndrome complicating systemic juvenile idiopathic arthritis: a multinational, multicenter study of 362 patients. Arthritis Rheumatol. 2014;66(11):3160-3169. doi:10.1002/art.38802

2. Baldo F, Erkens RGA, Mizuta M, et al. Current treatment in macrophage activation syndrome worldwide: a systematic literature review to inform the METAPHOR project. Rheumatology (Oxford). 2025;64(1):32-44. doi:10.1093/rheumatology/keae391

3. Shakoory B, Geerlinks A, Wilejto M HLH/MAS task force, et alThe 2022 EULAR/ACR points to consider at the early stages of diagnosis and management of suspected haemophagocytic lymphohistiocytosis/macrophage activation syndrome (HLH/MAS)Annals of the Rheumatic Diseases 2023;82:1271-1285.

